# Extra-Visual Functional and Structural Connection Abnormalities in Leber's Hereditary Optic Neuropathy

**DOI:** 10.1371/journal.pone.0017081

**Published:** 2011-02-10

**Authors:** Maria A. Rocca, Paola Valsasina, Elisabetta Pagani, Stefania Bianchi-Marzoli, Jacopo Milesi, Andrea Falini, Giancarlo Comi, Massimo Filippi

**Affiliations:** 1 Neuroimaging Research Unit, Scientific Institute and University Ospedale San Raffaele, Milan, Italy; 2 Division of Neuroscience, Department of Neurology, Institute of Experimental Neurology, Scientific Institute and University Ospedale San Raffaele, Milan, Italy; 3 Department of Ophthalmology, Scientific Institute and University Ospedale San Raffaele, Milan, Italy; 4 Department of Neuroradiology, Scientific Institute and University Ospedale San Raffaele, Milan, Italy; Cornell University, United States of America

## Abstract

We assessed abnormalities within the principal brain resting state networks (RSNs) in patients with Leber's hereditary optic neuropathy (LHON) to define whether functional abnormalities in this disease are limited to the visual system or, conversely, tend to be more diffuse. We also defined the structural substrates of fMRI changes using a connectivity-based analysis of diffusion tensor (DT) MRI data. Neuro-ophthalmologic assessment, DT MRI and RS fMRI data were acquired from 13 LHON patients and 13 healthy controls. RS fMRI data were analyzed using independent component analysis and SPM5. A DT MRI connectivity-based parcellation analysis was performed using the primary visual and auditory cortices, bilaterally, as seed regions. Compared to controls, LHON patients had a significant increase of RS fluctuations in the primary visual and auditory cortices, bilaterally. They also showed decreased RS fluctuations in the right lateral occipital cortex and right temporal occipital fusiform cortex. Abnormalities of RS fluctuations were correlated significantly with retinal damage and disease duration. The DT MRI connectivity-based parcellation identified a higher number of clusters in the right auditory cortex in LHON vs. controls. Differences of cluster-centroid profiles were found between the two groups for all the four seeds analyzed. For three of these areas, a correspondence was found between abnormalities of functional and structural connectivities. These results suggest that functional and structural abnormalities extend beyond the visual network in LHON patients. Such abnormalities also involve the auditory network, thus corroborating the notion of a cross-modal plasticity between these sensory modalities in patients with severe visual deficits.

## Introduction

Leber's Hereditary Optic Neuropathy (LHON) is a maternally inherited genetic disease characterised by an acute or subacute bilateral loss of vision, which predominantly affects young men, with a clinical onset between 15 and 35 years [1], [2], [3]. Pathologically, retinal ganglion cell degeneration and axonal loss in the optic nerve have been described in these patients [4]. These abnormalities are associated with an early and selective damage of the small calibre fibers of the papillomacular bundle. LHON has been linked to three “primary” mitochondrial DNA (mtDNA) point mutations, which affect oxidative phosphorylation in mitochondria [5], [6].

At present, it is still unclear whether central nervous system (CNS) involvement in patients with LHON is restricted to the optic nerve and visual pathways via chronic damage (i.e., the lateral geniculate nucleus and the visual cortex may be involved by trans-synaptic degeneration phenomena), as has been described in other ocular pathologies, including optic neuritis [7], [8], chronic glaucoma [9], retinal degeneration [10], and albinism [11]. Against this view militates the well-known association of LHON with clinical and magnetic resonance imaging (MRI) patterns indistinguishable from those of multiple sclerosis [12], as well as clinical observations which reported neurological disturbances, such as reflex alterations, cerebellar ataxia, periferic neuropathy and myoclonus in a relatively small percentage of these patients [13]. In addition, MR spectroscopy (MRS) studies of LHON have shown an abnormal mitochondrial energy metabolism in the occipital lobe [14], [15], [16], and diffuse abnormalities in the normal-appearing white matter have been detected using magnetization transfer MRI [17].

Functional MRI (fMRI) is a non-invasive technique which allows to define how the principal brain systems function in healthy subjects and to interrogate their alterations in patients with CNS pathologies. A method that has been introduced recently for the analysis of functional connections and coherence between different brain neural networks is based on the assessment of low-frequency (<0.1 Hz) fluctuations seen on fMRI scans acquired at rest (i.e., in the absence of any external stimulation). The use of such an approach has demonstrated the presence of a high temporal coherence between spatially distinct, functionally-related brain regions, resembling specific neuroanatomical networks, including the motor, visual, and dorsal and ventral attention systems, which characterise the resting-state networks (RSNs) of the human brain [18], [19], [20], [21]. The main advantage of RSN analysis is that it is not influenced by task performance and clinical impairment, as is the case for task-related fMRI.

In this study, we used fMRI to assess abnormalities within the principal brain RSNs in patients with LHON with the aim to define whether functional CNS abnormalities in this disease are limited to the visual system, or, conversely tend to be more diffuse and involve additional networks. In this latter case, to identify the possible anatomical substrates underlying the observed fMRI changes, an additional analysis was pre-planned, based on the exploitation of structural connectivity profiles of brain regions with significantly different activities between LHON patients and controls, using a connectivity-based analysis of diffusion tensor (DT) MRI data [22], [23], [24].

## Results

### Neuro-ophthalmologic assessment

Table 1 summarises the results of the neuro-ophthalmologic assessment in LHON patients. At the time of MRI assessment, all LHON patients had bilateral visual impairment and a variable degree of optic nerve pallor detectable at fundoscopy, which was particularly evident in the temporal sector. Standardised automated perimetry (SAP) showed a central scotoma of variable size in all affected eyes. Optic coherence tomography (OCT) detected a thinning of peripapillary retinal nerve fiber layer thickness (PRNFL) in all affected eyes, especially in the temporal quadrant.

**Table 1:** Results of neuro-ophthalmologic assessment in patients with Leber's hereditary optic neuropathy.

	Left Eye	Right Eye
Mean visual acuity (range)	1.33 (3-0)	1.14 (3-0)
Average mean deviation (range) (dB)	−15.73 (−0.43 to −32.61)	−15.48 (−1.48 to −33.35)
Average PRNFL thickness (range) (µm)	56.9 (33.1–89.3)*	56.5 (38.1–80.4)*
Temporal PRNFL thickness (range) (µm)	36 (31–47)*	49 (19–95)*

Abbreviations: PRNFL =  peripapillary retinal nerve fiber layer, SD = standard deviation.

*Below normal (<5^th^ percentile) as compared with a database of age-matched control subjects. See text for further details.

### MRI assessment

None of the subjects had T2-hyperintense brain lesions.

#### a) RS networks

**Figure 1:**
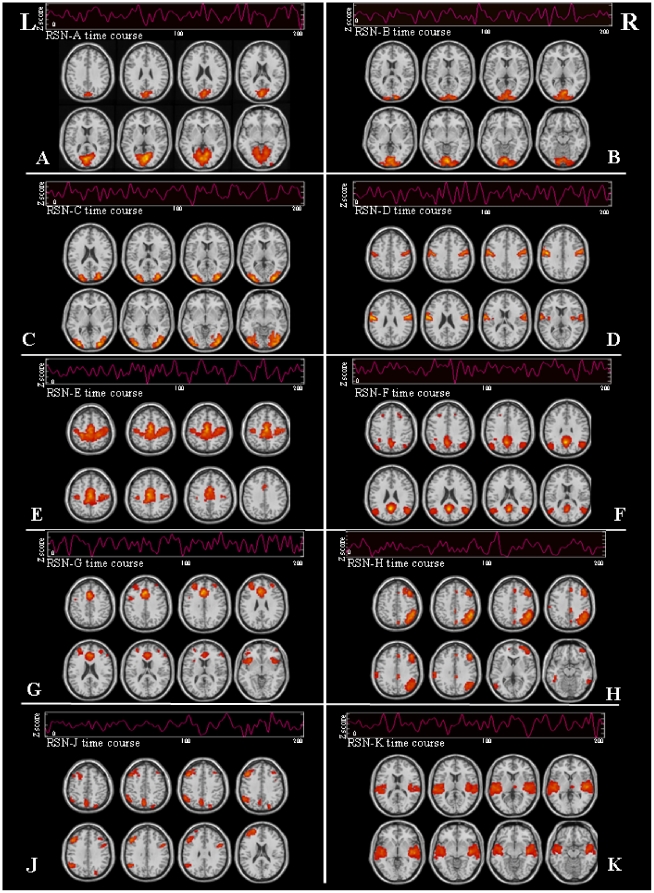
Spatial pattern and corresponding time courses of potentially functionally relevant resting state networks (RSNs) in healthy volunteers and patients with Leber's hereditary optic neuropathy: three RSNs included primary (A, B) and secondary (C) visual cortical areas; two RSNs (D, E) included sensorimotor areas; two RSNs (F, G) included areas that are part of the default mode network; two RSNs included fronto-parietal areas lateralized to the right (H) and left (J) hemispheres; one RSN (K) included primary and secondary auditory areas. See text for further details. Images are in neurologic convention.

In controls and LHON patients, the analysis of RS data detected 10 networks with potential functional relevance: three RSNs included the primary and secondary visual cortical areas, bilaterally; two the sensorimotor areas, bilaterally; two a set of cortical areas belonging to the default mode network (DMN); two a set of fronto-parietal areas lateralized to the right and the left hemisphere, respectively; and the last the primary and secondary auditory areas, bilaterally. Maps of RS activity for each of these networks, from both healthy controls and LHON patients (one-sample t test), are shown in Figure 1, together with their associated time courses. All these components were stable across multiple runs of IC decomposition, with a stability index assessed by ICASSO ranging from 0.72 to 0.98 for all the components of interest. RS networks of interest derived from the two ICA analyses performed on controls and LHON patients, separately, were very similar to those obtained from ICA performed on the entire study group (spatial correlation coefficients between networks ranging from 0.74 to 0.92).

#### b) Visual RS networks

**Figure 2:**
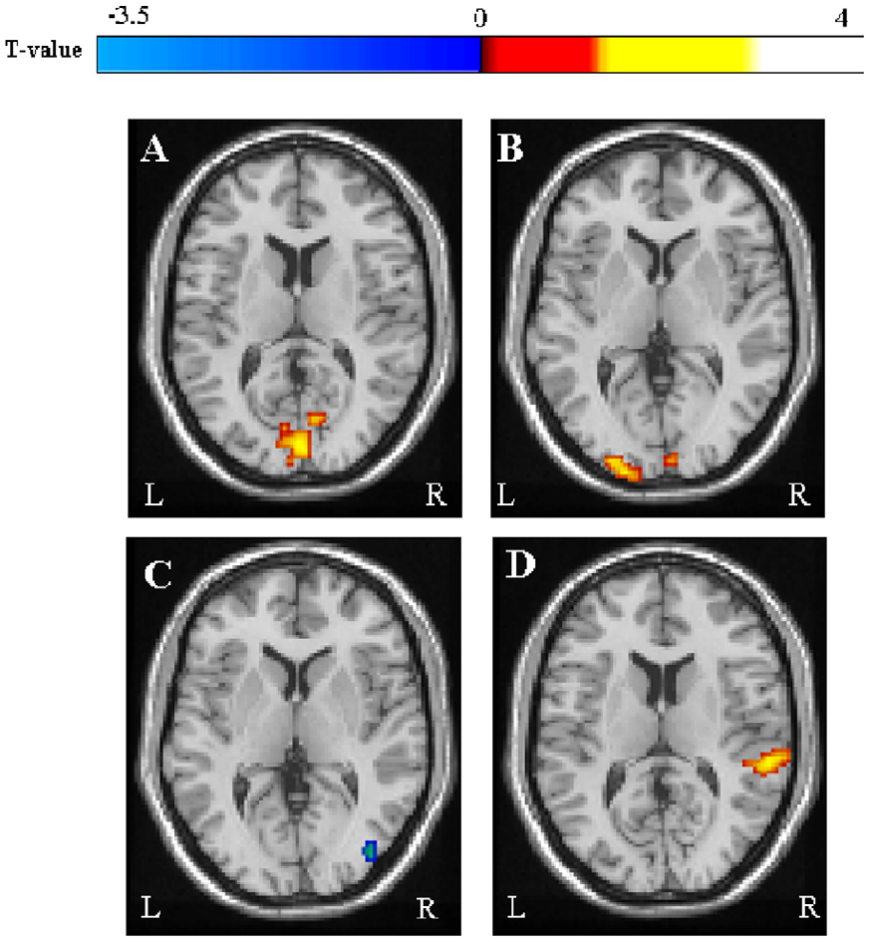
Spatial patterns of between-group differences in resting state (RS) fluctuations of the primary visual network (A, B), the secondary visual network (C) and the auditory network (D) between LHON patients and healthy controls. Clusters of increased RS activity in LHON patients are color-coded with red to yellow T values, while clusters of decreased RS activity in LHON patients are color-coded with dark to light blue T values. Images are in neurologic convention.

Between-group comparisons of the activity over the spatial extent of visual RSNs showed an increase of RS fluctuations in LHON patients *vs*. controls in several areas of the visual cortex in both primary visual RSNs. In detail, foci of increased RS fluctuations in the first visual network were located in the left cuneal cortex (MNI space coordinates: −3, −87, 20; t-value = 3.7; p = 0.05; cluster extent [k] = 5) and right supracalcarine cortex (MNI space coordinates: 3, −87, 12; t-value = 3.6; p = 0.05; k = 5). For the second visual network, the foci of increased RS fluctuations were located in the bilateral occipital pole (MNI space coordinates: 6, −93, −12; t-value = 4.6; p = 0.05; k = 5, and −30, −96, 0; t-value = 4.4; p = 0.05; k = 17), and the left occipital fusiform gyrus (MNI space coordinate: −15, −90, −12; t-value = 4.5; p = 0.05; k = 12) (Figure 2). This analysis also showed decreased RS fluctuations in the right lateral occipital cortex (MNI space coordinates: 45, −63, −8; t-value = 4.1; p = 0.05; k = 7), and right temporal occipital fusiform cortex (MNI space coordinates: 30, −54, −8; t-value = 3.8; p = 0.05; k = 5) for the network of the secondary visual areas (Figure 2).

#### c) Non visual RS networks

Between-group comparisons of the activity over the spatial extent of the non-visual networks showed significant increased RS fluctuations in the right superior temporal gyrus (including the primary auditory cortex) and the supramarginal gyrus (MNI space coordinates: 60, −33, 12: t-value = 4.6; p = 0.05; k = 11) for the RSN including the primary and secondary auditory areas (Figure 2). Using an uncorrected statistical threshold of 0.01, the same abnormalities were detected also in the left hemisphere. No between-group difference was found for the remaining networks/clusters.

#### d) Correlations of fMRI abnormalities with clinical and neuro-ophthalmologic measures

In LHON patients, significant correlations were found of disease duration with RS activity of the right lateral occipital cortex (r = −0.77, p = 0.01), left cuneal cortex (r = 0.87, p = 0.003), right occipital pole (r = 0.87, p = 0.003), and right superior temporal gyrus (r = 0.83, p = 0.02). In addition, significant correlations were found of average temporal PRNFL with RS activity of the left cuneal cortex (r = 0.88, p = 0.002) and right superior temporal gyrus (r = 0.79, p = 0.05).

#### e) DT MRI connectivity-based parcellation

**Figure 3:**
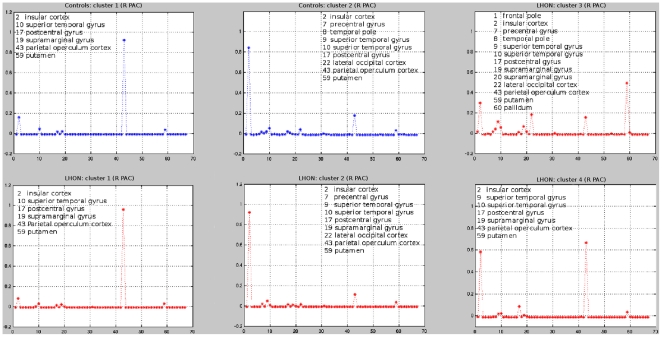
Clusters and cluster-centroids identified in the right (R) primary auditory cortex (PAC) in healthy controls (blue) and Leber's hereditary optic neuropathy (LHON) patients (red). The silhouette analysis identified two clusters in healthy controls and four clusters in LHON patients. The cluster-centroid profile analysis of the R PAC of LHON patients showed a structural connectivity with the frontal pole, the pallidum, and the supramarginal gyrus, which was not detected in controls. X-axis reports brain areas classified according to the Harvard-Oxford cortical and subcortical atlas.

The number of seed points that were used for tractography in each of the considered areas did not differ between controls and LHON patients. However, the number of seed points was significantly different between the left and the right auditory cortices, due to their different representation in the SPM Anatomical Toolbox [25]. The silhouette analysis identified a similar number of clusters in patients and controls for the right V1 (8 clusters in each group), the left V1 (8 clusters in each group), and the left auditory cortex (7 clusters in each group). Conversely, LHON patients had a higher number of clusters in the right auditory cortex compared to controls (4 clusters in patients *vs*. 2 clusters in controls) (Figure 3).

**Figure 4:**
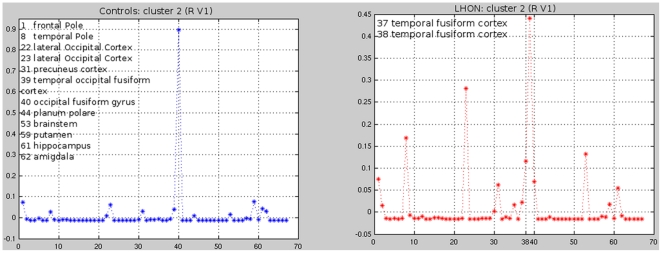
Cluster-centroid profile analysis of the right V1 (cluster II) in healthy controls (blue) and patients with Leber's Hereditary Optic Neuropathy (LHON) (red). The right V1 of LHON patients had a structural connectivity with the temporal fusiform cortex, which was not detected in controls. In addition, compared to controls, the right V1 of LHON patients had an increased structural connectivity with the temporal occipital fusiform cortex and a decreased structural connectivity with the lateral occipital cortex and the occipital fusiform gyrus. X-axis reports areas classified according to the Harvard-Oxford cortical and subcortical atlas.

The between-group differences in profile analysis are summarised in Table 2. The cluster-centroid profile analysis showed that the right V1 of LHON patients had a structural connectivity with the temporal fusiform cortex, which was not detected in controls (Figure 4). This analysis also showed that, compared to controls, the right V1 of LHON patients had an increased structural connectivity with the temporal occipital fusiform cortex and a decreased structural connectivity with the lateral occipital cortex and the occipital fusiform gyrus (Figure 4). The left V1 of LHON patients had a structural connectivity with the middle temporal gyrus and inferior temporal gyrus, which were not detected in controls. In addition, compared to controls, the left V1 of LHON patients had an increased structural connectivity with the frontal pole and lateral occipital cortex as well as a decreased structural connectivity with the temporal pole. The right auditory cortex (Figure 3) of LHON patients had a structural connectivity with the frontal pole, the pallidum, and the supramarginal gyrus, which were not detected in controls. Finally, the left auditory cortex of LHON patients had a structural connectivity with the lateral occipital cortex, which was not detected in controls. Conversely, controls had a structural connectivity with the middle temporal gyrus and inferior temporal gyrus, which was not detected in patients. Compared to controls, LHON patients had a lower number of seeds assigned to cluster 2 in the auditory cortex (Figure 3) (mean numbers of seeds [SD]: 8.1 [4.9] in LHON patients *vs*. 16.7 [5.9] in controls, p<0.001).

**Table 2:** Differences in cluster-centroid profiles between patients with Leber's hereditary optic neuropathy and healthy controls.

Areas	R V1	L V1	R Auditory cortex	L Auditory cortex
Temporal fusiform cortex	LHON only	-	-	-
Temporal occipital fusiform cortex	Increased LHON	-	-	-
Lateral occipital cortex	Decreased LHON	Increased LHON	-	LHON only
Occipital fusiform gyrus	Decreased LHON	-	-	-
Middle temporal gyrus	.	LHON only	-	Controls only
Inferior temporal gyrus	-	LHON only	-	Controls only
Frontal pole	-	Increased LHON	LHON only	-
Temporal pole	-	Decreased LHON	-	-
Pallidum	-	-	LHON only	-
Supramarginal gyrus	-	-	LHON only	-

Abbreviations: LHON = Leber's hereditary optic neuropathy, R = right, L = left, V1 = primary visual cortex.

In the left auditory cortex, significant correlations were found between:

- the number of seeds assigned to a cluster connected to the superior temporal gyrus *vs*. left temporal PRNFL (r = 0.63, p = 0.02) and average PRNFL (r = 0.61, p = 0.04);
- the number of seeds assigned to a cluster connected to the insular cortex and the parietal operculum cortex *vs*. temporal PRNFL (r = 0.62, p = 0.02).

#### f) Correspondence between areas of altered functional and structural connectivities

In LHON patients, a correspondence was found between:

- decreased functional connectivity in the right temporal occipital fusiform cortex and increased structural connectivity between the right visual cortex and the right temporal occipital fusiform cortex;
- decreased functional connectivity in the right lateral occipital cortex and decreased structural connectivity between the right V1 and the previous region;
- increased functional connectivity in the right supramarginal gyrus and increased structural connectivity between the right auditory cortex and the previous region.

## Discussion

We investigated RS fluctuations in patients with LHON to improve our understanding of this disease pathophysiology. The main advantage of the approach we used is that it does not require any active task to be performed, therefore its results are not influenced by possible between-group differences related to impairment and disability or to eye movements (which require an accurate monitoring when dealing with visual stimulations). As a consequence, we believe that these results can be considered representative of the actual cortical reorganization following tissue injury in LHON.

We detected 10 RSNs, which were consistently present in both controls and LHON patients. These RSNs included the sensorimotor areas, the primary and secondary visual systems, the auditory system, as well as attention and memory related networks. The between-group comparisons of the three visual networks showed increased fluctuations in regions that are part of the primary visual cortex, as well as decreased fluctuations in the right lateral occipital cortex and temporal occipital fusiform cortex in LHON patients in comparison to healthy controls, which might reflect a functional disconnection between primary and secondary areas within the visual network. Several studies, in patients with different ocular and retinal conditions have shown consistently that damage to the most anterior portions of the visual pathways is associated with functional and structural changes to the striate and extrastriate cortices, which are most likely secondary to trans-synaptic degeneration. A volume reduction of the visual cortical regions has been detected in patients with amblyopia [26], albinism [11], and retinal damage [10], [27]. A positron emission tomography study has demonstrated a high level of energy metabolism, at rest, in the visual cortex of early blind adults compared to age-matched blindfolded controls [28]. Our findings, combined with the results of these previous studies, suggest that synapses in the visual cortex of LHON patients are in a hyperactive state. Alternatively, they might reflect a variation of synaptic density. However, such an hypothesis is in contrast with the demonstration of a selective atrophy of the visual cortex in LHON patients [29].

Another important finding of our study is the demonstration that RSN abnormalities in LHON patients are not limited to the visual network, but also involve the auditory network. The coherence of activity between the visual and the auditory networks suggests the existence of an interplay between the two, which corroborates the notion of a cross-modal plasticity involving these sensory modalities in patients with severe visual deficits. Indeed, previous functional imaging studies have demonstrated that visual areas in blind subjects are activated by auditory tasks [30], [31]. Using a different method of analysis of low frequency RS fluctuations, based on the assessment of correlation coefficients between a large sets of brain regions, Liu et al. have recently shown decreased functional connectivities in the occipital visual cortex as well as between the visual cortices and the frontal, parietal and temporal cortices in early blind subjects (loss of sight at birth or before one year of age) in comparison to sighted individuals [32]. Differences in the methods of analysis as well as in the clinical and neuro-ophthalmologic characteristics of the patients included (early *vs*. late blindness) might contribute to explain the discrepancies between our and the previous [32] findings. In detail, we applied spatial ICA, which allowed us to cluster brain RS activity into different networks of spatially independent and temporally coherent brain regions according to the similarity of the reference time courses. The Z-scores of our spatial RS maps can be thought as a measure of intrinsic RS activity at a given voxel, but they do not give information about the similarity of such an activity with that of voxels from other distant regions belonging to other networks. On the contrary, the functional connectivity approach used by Liu et al. [32] investigated the correlations between pairs of brain regions. Such a method can detect significant couplings between time courses of spatially remote brain regions, but it does not provide any information about the level of RS activity of individual regions. Alternative applications of ICA, such as temporal ICA, might be used to obtain pieces of information more similar to those provided by functional connectivity, but temporal ICA is rarely used is functional neuroimaging literature, probably because of the computational challenges created by the higher data dimensionality [33].

Concerning the age of the onset of blindness, several studies have indicated a dramatic change in density of visual synapses during normal development, characterised by an increase between 2 and 8 months of age, and then by a decline (synaptic revision) to reach the adult level at 11 years [34], [35]. This synaptic revision corresponds to the elimination of redundant connections and to the establishment of functional connectivities. Since visual input interruption occurs prior to the stage of synaptic revision in early blind and after its establishment in late blind subjects, this might further contribute to explain the discrepancies between the two studies. Clearly, if confirmed by further work, our findings might have important therapeutic implications because they suggest that LHON patients might benefit from substitutive sensory aids. Remarkably, a recent gene expression profile study has shown that the optic atrophy 1 (OPA1) gene, which is related to autosomal dominant optic atrophy (ADOA), the most common form of hereditary optic neuropathy, is downregulated in some LHON patients [36]. Of note, OPA1 is expressed not only in the optic nerves and in the brain, but also in the inner ear.

To explore possible alterations of structural connections related to the above RS abnormalities, we performed a DT MRI connectivity-based parcellation analysis [22], which allowed us to investigate the structural connections between the primary visual and auditory cortices (which showed significant abnormalities of their functional connections) and other regions of the brain identified using a standard atlas. We choose such an approach, among the different strategies available to investigate the structural architecture of the WM using DT MRI, because the network of anatomical connections linking the neuronal elements of the human brain is still largely unknown, and we did not have strong a priori hypotheses on the possible anatomical connections to be investigated (with the exception of the optic tracts and radiations). A valid alternative strategy would have been the use of fMRI results to guide tractography reconstruction. However, with such a method we would have limited our results to a few WM tracts.

Our DT MRI connectivity-based parcellation analysis results need to be interpreted with caution, but nonetheless provide some important clues to better understand the RS fMRI changes seen in LHON patients. Consistently with the fMRI results which showed abnormalities of function within the auditory network (especially on the right side), the silhouette analysis identified a different number of clusters in healthy controls and LHON patients in the right auditory cortex. Furthermore, subtle differences of cluster-centroid profiles were found between the two groups for all the four seeds analyzed. Noteworthy, similarly to functional connectivity results, also the structural connectivity analysis disclosed areas of increased as well as decreased connectivity in patients *vs*. controls. Intriguingly, for a few of the areas identified by the two analyses, a correspondence was found between abnormalities of functional and structural connectivities. Remarkably, in one of these associations (i.e., decreased functional connectivity in the right temporal occipital fusiform cortex and increased structural connectivity between the right visual cortex and the right temporal occipital fusiform cortex) abnormalities of functional and structural connectivity had an opposite direction. Although we admit that this is only speculative, this observation suggests that structural and functional changes associated to the disease might be dynamic. A “temporal dissociation” between the two phenomena might explain this counter intuitive finding: abnormalities of functional connectivity might be a consequence of retinal damage, and might be then followed by an increased structural connectivity as an adaptive compensatory response.

Clearly, we can not define whether the abnormalities of WM architecture and function we detected in LHON patients are congenital or secondary to damage to the optic nerve. However, the extent of RS and DT MRI abnormalities observed in LHON patients was related to retinal damage (quantified using OCT) and disease duration, supporting the notion that they are likely to be the consequence of their deafferentation, following neuroaxonal damage to the retina and optic nerve.

Future investigations should evaluate whether the abnormalities we observed are stable or, conversely, whether they change over time, the factors influencing these changes, and, finally, whether they are specific of LHON or shared among other forms of hereditary optic neuropathy. This would help to define whether the analysis of RS connectivity might offer clinically relevant biomarkers of disease severity and duration in LHON patients.

## Materials and Methods

The study was approved by the Ethics Committee of Scientific Institute and University Ospedale San Raffaele, Milan, Italy and a written informed consent was obtained from all subjects prior to study entry, according to the Declaration of Helsinki.

### Subjects

We studied 13 patients with LHON (11 men and 2 women; mean age = 35.6, range = 20–61 years; mean disease duration = 9, range = 2–34 years) recruited from the Neuro-Ophthalmology Clinic at San Raffaele Scientific Institute, Milan, Italy. Inclusion criteria were: (i) presence of one of the three primary mtDNA mutations associated with LHON, (ii) disease duration >12 months, (iii) no history of concomitant neurological, psychiatric, ophthalmological diseases or drug abuse. Eight patients carried the 11778, three the 3460, and two the 14484 mtDNA mutation. Thirteen sex- and age-matched healthy subjects, with no history of neurological and ophthalmological disorders served as controls (11 men and 2 women; mean age 35.2, range 19–59 years).

### Neuro-ophthalmologic assessment

LHON patients underwent a complete neuro-ophthalmologic examination at the time of the enrolment. Best-corrected visual acuity was assessed with LogMAR notation performed with high-intensity red-free light. Visual field examination was performed with SAP and mean deviation was quantified (Humphrey Zeiss, 30-2 SITA standard program). Average and temporal PRNFL measurements were obtained using a commercially available optical coherence tomographer as previously described (Stratus OCT, Carl Zeiss Ophthalmic Systems Inc, fast RNFL thickness 3.4) [29].

### MRI acquisition

On a 3.0 Tesla Philips Intera scanner, RS fMRI scans were acquired within 24 hours from neuro-ophthalmologic assessment using a T2*-weighted single-shot echo planar imaging (EPI) sequence (repetition time [TR] = 3000 ms, echo time [TE] = 35 ms, flip angle = 90°, field of view [FOV] = 240 mm^2^; matrix = 128×128, slice thickness = 4 mm, 200 sets of 30 contiguous axial slices, parallel to the anterior-posterior commissural plane). Total acquisition time was about 10 minutes. During scanning, subjects were instructed to remain motionless, to close their eyes and not to think to anything in particular. All subjects reported they had not fallen asleep during scanning. Movements were minimised using foam padding and ear blocks.

A dual-echo turbo spin echo (TSE), a pulsed-gradient SE EPI (with diffusion gradients applied in 35 non-collinear directions; b factor = 900 mm^2^/s and a single b0 image), and a 3D high-resolution T1-weighted fast field echo (FFE) sequence were also obtained, as previously described [37].

### RS fMRI analysis

RS fMRI data were first pre-processed using Statistical Parametric Mapping (SPM5). All images were realigned to the first one to correct for subject motion (mean cumulative translations: 1.5 mm, SD = 0.27, and 1.9 mm, SD = 0.19 for controls and LHON patients, p = n.s.; mean rotation <0.2 degrees in both groups). The mean individual motion was calculated for each subject as the average of the six realignment parameters estimated by SPM5. Data were then spatially normalised into the standard Montreal Neurological Institute (MNI) space (with a sub-sampling to 3×3×4 mm^3^ resolution, leading to images with a matrix = 53×63×35, and, therefore, a total number [N] of voxels = 116865), using the standard SPM5 EPI template as a reference, and smoothed with a 6-mm, 3D-Gaussian filter. Linear detrending and band-pass filtering between 0.01 and 0.08 Hz were performed using the REST software (http://resting-fmri.sourceforge.net/) to partially remove low-frequency drifts and physiological high-frequency noise.

Independent Component Analysis (ICA) was used to decompose RS fMRI data into spatially independent maps and time courses using the GIFT software (Group ICA of FMRI Toolbox) [33]. GIFT analysis was performed following three main steps: (i) data reduction, (ii) ICA, and (iii) back reconstruction. First, single-subject fMRI data were reduced to a lower temporal dimensionality by using a principal component analysis. The number of independent group components was 40, a dimension determined using the minimum description length criterion [33]. Then, for each time-component of each subject, the 3D fMRI image was flattened to a 1D vector (with dimension N = 116865 voxels) and a single-subject 40×N matrix was created, containing all flattened images for the 40 temporal components. Finally, the matrices from each of the 26 subjects were vertically concatenated into a M×N multi-subject matrix (M = 26×40 = 1040) containing the fMRI data from all subjects. The group independent components (ICs) were estimated using the Infomax approach [38], and each component was represented by a spatial map and a temporal profile. The group mixing matrix estimated by Infomax, which describes group ICs, can be also thought as made by many side-by-side partitions, each of which is related to ICs of a single subject. Individual subject components maps and time courses were back-reconstructed by subdividing group ICs into the corresponding single subject partitions [33]. Statistical reliability of IC decomposition was tested by using the ICASSO toolbox [39]. Stability of the obtained ICs was assessed by running Infomax 10 times with different initial conditions and bootstrapped datasets, by clustering the results of each run, and by calculating a stability index for each component. Two separate spatial ICAs were also performed in controls and LHON patients to ensure that the resulting components were similar in the two groups. Similarity was assessed by calculating the spatial correlation coefficient between components estimated by each ICA analysis, using the “fslcc” utility included in FSL toolbox (http://www.fmrib.ox.ac.uk/fsl/).

Each individual functional map was converted into Z-scores before entering group statistics, to have voxel values comparable across subjects.

A systematic process was applied to inspect and select the components of interest from the 40 estimated components. The association of each component spatial map with a priori probabilistic maps of gray matter (GM), white matter (WM), and cerebrospinal fluid (CSF) within the MNI space contributed to identify the components with a signal change correlated to the GM. Components with a high correlation to CSF or WM, or with a low correlation with the GM, were excluded. In addition, to identify components with potential functional relevance, a frequency analysis of IC time courses was performed to detect those with a high (50% or greater) spectral power at a low frequency (between 0.01 and 0.05 Hz) [40]. The spatial patterns of the remaining ICs were sorted out on the basis of their matching with relevant RSNs found in previous studies [18], [19], [20], [21].

The magnitude of RS activity within each group, as well as between-group RS activity differences were assessed using SPM5 and a one-sample t test and a two-sample t test, respectively, including the mean subject's motion (calculated as the mean of the six motion parameters estimated by SPM) [41], [42] as a confounding covariate. SPMs produced at one-sample t tests were thresholded at p = 0.001 and combined (intersection) in a single image, which was used as mask for the subsequent between-group comparisons. Results of between-group comparisons were then superimposed to the Harvard-Oxford cortical atlas (http://www.fmrib.ox.ac.uk/fsl/fslview/atlas-descriptions.html#ha) to have the results in the same atlas of the structural connectivity results.

In LHON patients, using SPM5 and multiple regression models, a linear regression analysis was performed to assess spatial correlations of RS abnormalities with clinical and neuro-ophthalmologic measures. Clusters exceeding a threshold of p<0.001 (uncorrected for multiple comparisons) underwent a small volume correction (SVC) for multiple comparisons, setting the cut off value for significance at p<0.05 and using a 10-mm radius around the peak derived from the between-group comparisons.

We used a family wise error (FWE) correction at p<0.05 for multiple comparisons at a cluster level as the threshold for statistical significance for between-group comparisons. Only clusters having an extent of k≥5 were included in this analysis. This extent should not be considered small, because RS data, due to image subsampling to 3×3×4 mm ( = 36 cubic mm volume of each voxel), have a single voxel volume equivalent to that of 4.5 voxels of active fMRI data pre-processed with a standard SPM5 analysis, having usually a dimension of 2×2×2 mm ( = 8 cubic mm volume).

### DT MRI connectivity-based parcellation analysis

DT images were first corrected for distortion induced by eddy currents using an in-house software [43]. The DT was then calculated on a pixel-by-pixel basis, using FSL tools (http://www.fmrib.ox.ac.uk/fsl).

Based on the results of RS fMRI analysis (see above), a parcellation analysis was performed on the primary visual (V1) and auditory cortices, bilaterally. They were segmented on 3D T1-weighted images using an atlas-based approach. To this aim, V1 and the auditory cortex (Areas TE 1.0, TE 1.1 and TE 1.2) [44] were selected using the SPM Anatomy Toolbox [25], normalised, using SPM5, to single-subject 3D T1-weighted images (which were previously scalped using the Brain Extraction Tool [BET] [45] and coregistered to the b0 images), and thresholded at 50%. Seeds for tractography were selected as follows: 3D T1-weighted images were segmented into WM, GM and CSF using SPM5 and registered to diffusion space; then, a 2D sobel filter was applied to the GM maps (not binarized) to obtain the boundaries between GM and WM. Finally, the resulting contours were masked with the visual and auditory cortical probability maps derived previously; seeds facing the CSF were removed manually. The resulting seeds were used as starting points for probabilistic tractography [24]. The output of tractography is a file containing the number of visits of tracts in each voxel; this is considered as an index of structural connectivity. Due to computational demands, this output is saved at a 5 mm resolution [23]. Connectivity values from tractography were then summed up within cortical regions identified with the Harvard-Oxford cortical and subcortical atlas available within FSL (http://www.fmrib.ox.ac.uk/fsl/fslview/atlas-descriptions.html#ha). The regions containing the starting seeds were excluded. Connections to the contralateral hemisphere were not considered. Finally, data were reformatted in a matrix layout in which each row is the connectivity profile of a single seed to each of the considered cortical areas, and matrices for all subjects were concatenated in a single matrix, which is the input of the k-means algorithm for clustering. Since the k-means method requires as an input the number of clusters, we used the silhouette method [46], [47] to estimate the number of clusters that fitted best. The silhouette method is based on the production of a silhouette profile for each cluster found, which defines how good is the classification of each seed in comparison with its assignment to a second most appropriate cluster. For each seed, the profile plots an index, which is calculated from the ratio between the average similarity of the seed to all other seeds in the cluster and the maximum similarity of the seed to all seeds of other clusters. Index values close to 1 indicate a well-clustered seed, whereas values close to -1 indicate a bad assignment of the seed to a given cluster. Once all silhouettes are combined in a single plot, the average value can be used to select the most appropriate number of clusters.

To do this, we run silhouette for different number of clusters (ranging from 2 to 15) and calculated the average silhouette values for each trial. The optimal number of clusters was that corresponding to the maximum value of the average silhouette profile. During both the optimization and the clustering phase, the control and patient groups were treated separately. The clustering analysis also provided the cluster-centroids, which summarise the structural connectivity profile of each cluster. First, a visual inspection was performed to identify between-group differences in cluster-centroid profile (e.g., presence/absence of a given peak and peak amplitude). Then, for each subject, the number of seeds assigned to each cluster was used as a subject-wise measure to compare patients with controls (t test for independent samples), and to assess correlation with clinical and ophthalmological measures (non parametric correlation, Spearman Rank Correlation Coefficient).
